# Clinical characteristics of 9 cancer patients with SARS-CoV-2 infection

**DOI:** 10.1186/s13020-020-00328-8

**Published:** 2020-05-14

**Authors:** Yong Zeng, Bo Zhang, Xufeng Zhang, Cunjian Yi

**Affiliations:** 1 The First People’s Hospital Affiliated to Yangtze University in Jingzhou, 8 Hangkong Road, Jingzhou, Hubei China; 2 Shanghai Chest Hospital Affiliated To Shanghai Jiaotong University, Shanghai, China

**Keywords:** Cancer, SARS-CoV-2, Clinical characteristics, Outcomes

## Abstract

In December 2019, a cluster of pneumonia cases was caused by the Severe Acute Respiratory Syndrome coronavirus 2 (SARS-CoV-2) in Wuhan, China. Cancer patients are a special group, the immunity of them will be suppressed because of various anti-tumor treatments, and the risk of infection will be greatly increased, so we will report clinical features of 9 cancer patients with SARS-CoV-2 infection. 5 (56%) patients were ordinary type, 3 (33%) were severe type, and 1 (11%) was critical type. A total of 8 patients received combined therapy of traditional Chinese medicines and western medicines. From the clinical outcomes of these 8 patients, western combined therapy of traditional Chinese medicine was indeed an effective treatment method. D-dimmer rise, infection index rise, and chest CT(computed tomography) progression may be clinical warning indicators for severe patients, in our study, more 50% of patients had elevated levels of these indicators, but only 44% (including the dead) of patients had received treatment in the intensive care unit. 5 (56%) ordinary type patients had been discharged, while the 1 (11%) critical type patient died 3 days after admission. Cancer comorbidity seems to have no direct relationship with severe events, and the combination of traditional Chinese medicine and western medicine may be effective in the prevention and treatment of novel coronavirus-infected pneumonia (NICP).

## Background

In December 2019, a cluster of pneumonia cases caused by the SARS-CoV-2 occurred in Wuhan, Hubei province [1, 2]. Now it has developed into an international public health emergency. However, in this new outbreak of novel NCIP there is a special population that cannot be ignored—cancer patients. After receiving various anti-tumor treatments, the immunity of cancer patients will be suppressed, and the risk of infection will be greatly increased [3–6] .Liang found that cancer patients in SARS-CoV-2 infection had poorer outcomes [7]. However, neither Yang Xia [8] nor Hanping Wang [9] through there was a strong link between cancer and SARS-CoV-2 infection. Here, so we will report the clinical features of cancer patients with SARS-CoV-2 infection to provide further knowledge of this disease.

## Data collection

**Fig. 1:**
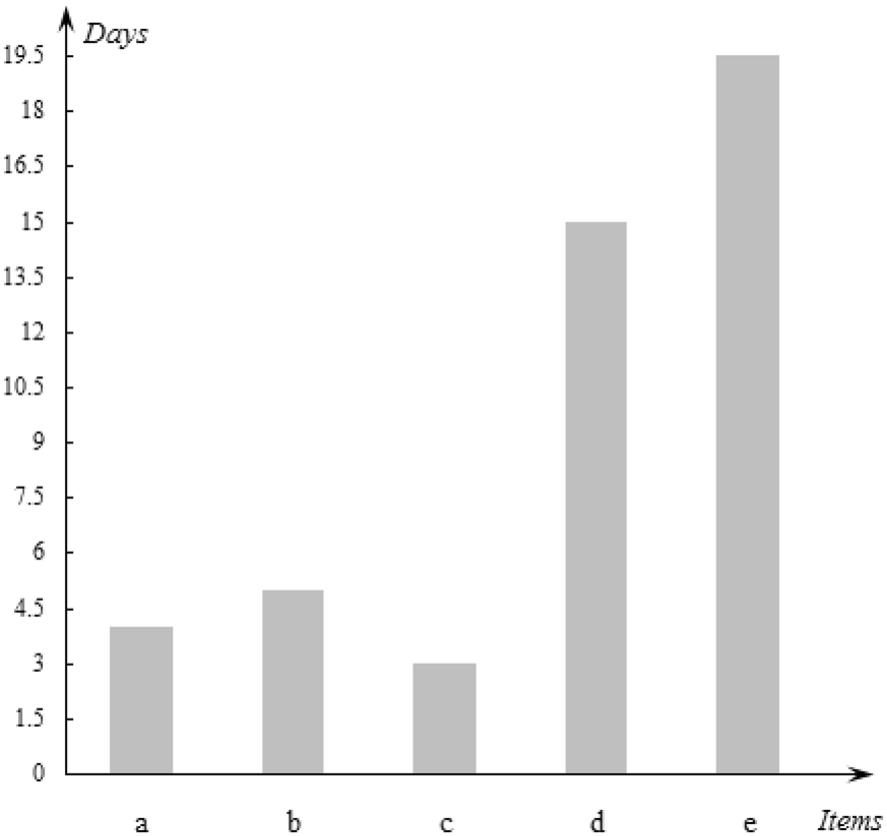
The median time from onset of symptoms: **a** to first hospital admission was 4 (1–15) days; **b** to 2019-nCoV positive was 5 (2–20)day; **c** to first chest CT images was 3 (1–10) days; **d**. to intensive care unit admission was 15 (2–23)days; **e** the median time from nCoV positive to negative was 19.5 (2–26)days

This study was approved by the institutional ethics board of Yangtze University. A retrospective, single-institutional review of cancer patients with SARS-CoV-2 infections was conducted between January 23 and February 29 2020, at the First People’s Hospital affiliated with Yangtze University in Jingzhou, China. Basic information, clinical manifestations, laboratory, medical imaging and outcome dates were collected from patients’ medical records and were exhibited in Additional file 1: Appendix 1, Additional file 2: Appendix 2, Additional file 3: Appendix 3 and Additional file 4: Appendix 4 respectively. The median time was exhibited in Fig. 1, included from onset of symptoms to SARS-CoV-2 positivity, from onset of symptoms to intensive care unit admission, from SARS-CoV-2 positive to negative. As of March 3, 2020, the novel coronavirus detection of 8 (100%) patients was negative. Outcomes were followed up until March 8, 2020.

## Treatment regimen

This disease belongs to the category of traditional Chinese medicine epidemic diseases, and caused by the plague poison. Studies [10] found that the combination of traditional Chinese medicine and western medicine was effective in the prevention and treatment of NICP in all stages, and the response rate of symptoms such as fever, cough and fatigue were significantly increased in ordinary patients after taking lianhua qingwen granules. In the critical stage, modern medicine approaches can play a powerful role in patients’ life support and the control of complications, xuebijing injection combination with modern medicine approaches can improve the cure rate. In addition, compared with the western medicine group, the chinese-western combined treatment group had shorter test negative conversion time, higher symptom improvement rate and shorter hospital stay. Therefore, the combination of Chinese medicine and western medicine was effective in the treatment of NICP. In our study, a total of 8 patients received combined therapy of traditional Chinese medicines and western medicines. 8 (89%) patients received oral antiviral treatment using Lianhua Qingwen granules and Arbidol. 3 severe patients were given additional intravenous Xuebijing injection. In addition, through the different stages of COVID-19 infection of the body dialectical, based on Lung Cleaning and Detoxifying Decoction, flexible use of Chinese medicine prescription. All patients were given antibiotics. 8 (89%) patients were given a combination of antibiotic treatments. 5 (56%) patients were given intravenous methylprednisolone. Immunoglobulin was provided to 3 (33%) patients (10–14 days). 7 (78%) patients were treated with oxygen inhalation (nasal catheter or mask), 4 (44%) were treated with high-flow oxygen inhalation, and one patient used non-invasive mechanical ventilation. From the clinical outcomes of these 8 patients, western combined therapy of traditional Chinese medicine was indeed an effective treatment method, but due to the limited case data, more clinical practice data were needed to evaluate.

## Clinical outcomes

Of the 9 patients, 5 (56%) patients were ordinary type, 3 (33%) were severe type, and 1 (11%) was critical type. 1 patient (critical type, 82 years old) died from multiple organ failure, and the interval time from onset of symptom to death was 3 days. 5 patients (ordinary type) had been discharged, and their median time of hospitalization was 26 (7–29) days. All other patients were still in hospital. According to the pneumonia diagnosis and treatment protocol for novel coronavirus infection released by the National Health Commission (version 6), D-dimmer rise, infection index rise, and chest CT progression may be clinical warning indicators for severe patients. In our study, a majority of patients had differing degrees of increased infection index, which may represent more prominent inflammation. 78% of patients had increased D-dimmer, and previous studies had found that D-dimmer increase was related to severity of illness [11, 12]. 56% of patients showed signs of multiple mottling and ground-glass opacity, in other studies, early-stage chest CT examination mostly showed multiple, small patch-like shadows and interstitial changes [13], which may indicate that in cancer patients with SARS-CoV-2 infections lung lesions progressed faster. But only 44% (including the dead) of patients had received treatment in the intensive care unit. Additionally, of the 5 cancer patients with SARS-CoV-2 infections receiving anti-tumor treatment in the last year, only 2 (40%) also had been diagnosed as severe type, with the other (60%) diagnosed as ordinary type. This seems to indicate that cancer comorbidity may have no direct relationship with severe events.

## Conclusion

By analyzing 9 cancer patients with SARS-CoV-2 infection, cancer comorbidity seems to have no direct relationship with severe events, and the combination of traditional Chinese medicine and western medicine may be effective in the prevention and treatment of NICP. So the study may offer some suggestions about nurse and treatment of cancer patients with SARS-CoV-2 infection.

## Supplementary information

**Additional file 1.** Cancer related history and treatment of cancer patients with 2019-nCov infection.

**Additional file 2: Table S1.** General information and Clinical manifestations of cancer patients with 2019-nCoV infection.

**Additional file 3: Table S2.** Laboratory dates of cancer patients with 2019-nCoV infection.

**Additional file 4: Table S3.** Chest CT images of cancer patients with 2019-nCoV infection.

## Data Availability

The datasets used and analysed during the current study are available from corresponding author on reasonable request.

## Abbreviations

SARS-CoV-2: Severe Acute Respiratory Syndrome coronavirus 2
NCIP: Novel coronavirus-infected pneumonia
CT: Computed tomography
COVID-19: Corona Virus Disease 2019
