# Supplementary material for: Clinical characteristics of 9 cancer patients with SARS-CoV-2 infection
Source: Chin Med. 2020 May 14;15:47. doi: 10.1186/s13020-020-00328-8 (PMC7224342; doi:10.1186/s13020-020-00328-8)
Supplement: Supplementary file 1 — Additional file 1. Cancer related history and treatment of cancer patients with 2019-nCov infection. [file 13020_2020_328_MOESM1_ESM.pdf]

Patients	anti-tumor treatment	Diagnosis
1	In 2011, she underwent surgery for thyroid cancer,no additional treatment was performed.	Ordinary
2	In July 2019, he underwent surgery for lung cancer, four times of postoperative chemotherapy,the last one in October 2019.	Ordinary
3	In 2015, she underwent surgery for breast cancer,Postoperative adjuvant therapy was not clear	Ordinary
4	In 2019, she underwent surgery for breast cancer, six times of postoperative chemotherapy, the last one in December 2019.	Severe
5	In December 2019, she underwent surgery for ovary cancer, no additional treatment was performed	Severe
6	In 2017, she underwent surgery for breast cancer, eight times of postoperative chemotherapy the last one in June 2019	Ordinary
7	She had breast cancer surgery 10 years ago， no additional treatment was performed.	Severe
8	In 2016, she underwent surgery for kidney cancer, in June2019, she underwent surgery for breast cancer， no additional treatment was performed.	Ordinary
9	Bladder cancer ,but his medical status was unclear	Critical
