# Supplementary material for: Clinical characteristics of 9 cancer patients with SARS-CoV-2 infection
Source: Chin Med. 2020 May 14;15:47. doi: 10.1186/s13020-020-00328-8 (PMC7224342; doi:10.1186/s13020-020-00328-8)
Supplement: Supplementary file 2 — Additional file 2: Table S1. General information and Clinical manifestations of cancer patients with 2019-nCoV infection. [file 13020_2020_328_MOESM2_ESM.pdf]

Table 1 General information and Clinical manifestations of cancer patients with 2019-nCoV infection

Items		n	%
Age	median(range)	66(51-85)years	
Gender			
	Female	7	78%
	Male	2	22%
Epidemiological			
	exposure to confirmed nCoV infection patient	5	56%
	Unknow	4	44%
Cancer type			
	Breast cancer	5	56%
	Bladder cancer	2	22%
	Lung cancer	1	11%
	Thyroid cancer	1	11%
	Ovary cancer	1	11%
Comorbidity			
	Hypertension	7	78%
	Other(diabetes,cerebral infarction)	3	33%
Symptom			
	Fever	8	89%
	Temperature median(range)	38℃(36.4℃---38.5℃)	
	<37.3℃	2	22%
	37.3℃--38℃	3	33%
	>38℃	4	44%
	Cough	3	33%
	Chills	2	22%
	Asthma	2	22%
	Headache	1	11%
	Fatigue	1	11%
No symptom		1	11%
Blood oxygen saturation %		96%(80%-99%)	
