# Supplementary material for: Clinical characteristics of 9 cancer patients with SARS-CoV-2 infection
Source: Chin Med. 2020 May 14;15:47. doi: 10.1186/s13020-020-00328-8 (PMC7224342; doi:10.1186/s13020-020-00328-8)
Supplement: Supplementary file 3 — Additional file 3: Table S2. Laboratory dates of cancer patients with 2019-nCoV infection. [file 13020_2020_328_MOESM3_ESM.pdf]

Table 2 Laboratory dates of cancer patients with 2019-nCoV infection

Items \ Patients	1	2	3	4	5	6	7	8	9
white bloold cell (3.95-9.5×10^{12}/L)	5.7	8.41	6.6	4.25	11.2	3.1	4.94	2.85	14.5
neutrophils (1.8-6.3×10^{9}/L)	4.25	4.38	3.07	4.7	9.61	2.43	3.92	1.68	12.4
lymphocyte (1.1-3.2×10^{9}/L)	1.03	3.3	2.66	1.28	0.94	1.47	0.68	0.85	1.71
alanine aminotransferase (1-40 U/L)	11	6	27	160	21	14	17	33	38
aspartame aminotransferase (2-42 U/L)	25	19	22	57	35	4	39	57	42
albumin (35-55 g/L)	35.3	44.4	40.4	34.3	30.1	33.9	35.8	42.5	40.7
lactate dehydrogenase (100-240 U/L)	217	244	174	317	447	148	320	257	399
serum creatinine (44-106 umol/L)	50.1	84.4	65.5	44.7	32	49.1	105	106	138
blood urea nitrogen (1.8-7 mmol/L)	2.86	4.59	6.57	3.19	2.59	2.18	8.97	7.98	11.6
prothrombin time (9.9-12.5 s)	10.8	11.7	10.3	10.8	10.9	10	11.5	10.9	10.2
activated partial thromboplastin time (23-38 s)	24.6	27.8	24.1	27.8	28.4	26.7	34.3	29.4	21.2
D-dimmer (0.00-0.55 mg/L)	0.67	0.2	0.48	0.73	3.19	12.7	2.44	1.25	80
C-reactive protein (0-8 mg/L)	31.6	0.77	1.39	56.6	45.6	1.4	93	13.3	232
erythrocytes sedimentation rate (0-15 mm/H)	90	35	73	60	54	30	124	28	79
troponin (0-34.2 pg/ml)	13.5	4.6	2.6	12.8	0.7	1.5	67.7	5	124
brain natriuretic peptide (0-100 pg/ml)	34.1	63.2	37.4	81.6	<10	25.6	250	75.1	105
