# Supplementary material for: Clinical characteristics of 9 cancer patients with SARS-CoV-2 infection
Source: Chin Med. 2020 May 14;15:47. doi: 10.1186/s13020-020-00328-8 (PMC7224342; doi:10.1186/s13020-020-00328-8)
Supplement: Supplementary file 4 — Additional file 4: Table S3. Chest CT images of cancer patients with 2019-nCoV infection. [file 13020_2020_328_MOESM4_ESM.pdf]

Table 3 Chest CT images of cancer patients with 2019-nCoV infection

Items	n	%
First chest CT imaging		
Duration from onset of symptoms to first chest CT image	3(1-10)days	
Unilateral pneumonia	3	33%
Bilateral pneumonia	6	67%
Multiple mottling and ground-glass opacity	5	56%
Pleural effusion	1	11%
Pleural thickening	2	22%
